# (De)Prescribing of proton pump inhibitors: what has changed in recent years? an observational regional study from the French health insurance database

**DOI:** 10.1186/s12875-022-01941-2

**Published:** 2022-12-30

**Authors:** Pauline Gendre, Julie Mocquard, Pascal Artarit, Anicet Chaslerie, Pascal Caillet, Jean-François Huon

**Affiliations:** 1 Pharmacy, Nantes Université, CHU Nantes, 44000 Nantes, France; 2 Medical Department, French National Health Insurance, DRSM, Nantes, France; 3 Public Health Department, Nantes Université, CHU Nantes, 44000 Nantes, France

**Keywords:** Deprescribing, Proton pump inhibitors, Epidemiology, Inappropriate prescribing

## Abstract

**Background:**

Proton pump inhibitors (PPIs) are one of the most widely prescribed drug classes in the community and at hospital. The significant misuse of PPIs requires the implementation for a deprescribing strategy. Numerous studies aiming at evaluating the impact of deprescribing interventions have been set up, implying a precisely known evolution of consumption of PPIs in the population studied without intervention.

The main objective of the study was to study overall changes in PPI prescribing and deprescribing in a regional population of chronic consumers without intervention, according to health insurance databases.

**Methods:**

This historical cohort study was based on the French National Health Data System databases. All adult patients living in the Pays de la Loire area and covered by the French National Health Insurance and who had at least one reimbursement for a PPI dispensing between 01 October 2016 and 31 December 2020 were included. Only chronic consumer patients were included, defined as patients who has had PPI dispensed for 3 consecutive months with a temporal coverage of at least 80%. Patients under 18 years of age and patients who received parenteral PPIs only were excluded.

**Results:**

The percentage of chronic treatment discontinuation in 2017 was 12.5% and remained stable to reach 12.4% in 2020. The number of new chronic patients increased from year to year to reach 77,222 patients in 2020, with an increasing rate of 1.2 to 2% between 2017 and 2020. The prevalent patient population increased from year to year to reach 167 751 patients in 2020, with an increasing rate of 4.2 to 4.4% between 2017 and 2020.

Regarding the initiation of PPI therapy, in 2020, 87.1% of treatment initiations were done by general practitioners. They renewed 2,402,263 prescriptions (89.3%) between 2017 and 2020.

**Conclusions:**

This study shows a stagnation over the last 4 years in the deprescribing of chronic PPI treatments in a French region despite the information on their inappropriate use reported by national agencies and in the literature with increasing frequency. This reinforces the interest of setting up a deprescribing project.

## Background

Proton pump inhibitors (PPIs) have been marketed since 1989 in France. They are one of the most widely prescribed drug classes in the community and at hospital. For a long time, PPIs were viewed as safe and well tolerated medications because of their specific action, but in recent years concerns regarding associated adverse events emerged [1, 2]. Even if mild side effects are considered to be the most frequent and generally reversible when treatment is stopped, more serious but rarer adverse outcomes may become significant in terms of public health in a population exposed to long-term use of these drugs [3–5].

PPIs consumption is growing. Between 2010 and 2015, PPI sales in France increased by approximately 27%, reaching more than 85 million boxes sold in 2015 [6]*.* More than half (54%, or nearly 4 million) of new adult users were starting PPI treatment to prevent or treat gastroduodenal lesions caused by non-steroidal anti-inflammatory drugs (NSAIDs). Among these, more than 90% started concomitantly NSAIDs, suggesting a preventive approach to the adverse effects induced by these treatments. Nevertheless, in 80% of cases, no patient risk factors justifying their use were found.

The significant misuse of PPIs requires the implementation for a strategy of deprescribing and the involvement of health professionals as well as patients themselves [7]. Deprescribing is a complex process, defined as reducing dose, stopping or using “on-demand” dosing, which directly and minimally involves the patient and the prescriber [8]. The decision to stop or reduce the use of PPIs must be based on information and understanding of the benefits and risks of continuing or stopping treatment [9]. Many methods can be used, but some show little effectiveness, such as issuing recommendations or educational programs [10].

Numerous studies aiming at evaluating the impact of deprescribing interventions have been set up at different levels, frequently local [11]. However, interpreting adequately their results or designing new ones on a larger scale implies knowing precisely the evolution of consumption of PPIs in the population studied without intervention. Therefore, before any large-scale deprescribing trial is implemented, we felt it necessary to evaluate the evolution of PPI consumption in a French regional context.

## Methods

The main objective of the study was to evaluate the overall evolution in PPI de-prescribing without intervention in a regional population of chronic consumers.

The secondary objectives were to describe the evolution of the incidence and prevalence of PPI consumption, the characteristics of prescriptions, of patients and of prescribers.

This study is an historical cohort study based on the French National Health Data System (SNDS) databases, including exhaustive national claims data for all individuals covered by the French National Health Insurance scheme between October 1, 2016 and December 31, 2020. The SNDS links several existing databases: the nationwide claims database of the French National Healthcare system, the national hospital database and the national death registry. The SNDS covers more than 98% of the French population (66 million people) from birth (or immigration) to death (or emigration), even in case of change in occupation or retirement. The SNDS contains a longitudinal record of health encounters, hospital diagnoses and drugs deliveries relative to outpatient medical care claims. The Pays de la Loire Region is socio-demographically representative of the French territory. Indeed, these are very strongly comparable in terms of average age, age categories, socio-professional categories, sex ratio, median income and access to general practitioners [12–16]. This makes it a suitable territory for a descriptive observational study that is then replicable. All adult patients (aged over 18 years) living in the Pays de la Loire area and covered by the French National Health Insurance (whatever the social security scheme) and who had at least one reimbursement for a PPI dispensing between 01 October 2016 and 31 December 2020 were included. Only chronic consumer patients were included.

### Definitions

- A patient was defined as a *chronic user* when he was dispensed at least 74 doses of PPI over 3 consecutive months, i.e. a temporal coverage of at least 80%. Thus, a patient was a *chronic user* when he was a PPI’s user on January 1st of year n, with drug exposure over the last 3 months (October to December) of year n-1. Patients under 18 years of age and patients who received parenteral PPIs only were excluded.
- A patient classified as a *chronic user* in year n but who was not a user on January 1 of year n-1 was considered as a *chronic incident* or *chronic new user*.
- Discontinuation of chronic status treatment was considered when a patient who was a *chronic user* in year n no longer used PPI (no more refill) during the last two months (November and December) of year n.
- After stopping treatment for at least 2 consecutive months, resumption of treatment was counted as a new episode of chronic use. The number of drugs delivered to a patient was defined either in units dispensed or in DDD (defined daily dose). The DDD is defined as the assumed average maintenance dose per day for a drug used in its primary indication in adults [17].

Data from the SNDS provided access to information about patient such as: age, gender and place of residence. The prescriber's medical specialty was categorized into 3 groups: general practitioner, specialist, or public health care institution physician.

According to data protection and the French regulation, the authors cannot publicly release the data from the French national health data system (SNDS). However, any person or structure, public or private, for-profit or nonprofit, is able to access SNDS data upon authorization from the French Data Protection Office (CNIL Commission Nationale de l’Informatique et des Libertés) to carry out a study, a research, or an evaluation of public interest.

The National Health Data System (SNDS) is a set of strictly anonymous databases, comprising all mandatory national health insurance reimbursement data. No informed consent was required because data are anonymized.

## Results

There was no missing data.

The socio-demographic characteristics of incident chronic consumer patients between 2017 and 2020 are described in Table 1. The age of the incident chronic users over the four years was 67.4 years on average. The proportion of women was 54.4%. The number of incident patients increased from year to year to reach 77,222 patients in 2020. The rate increased from 1.2% to 2% between 2017 and 2020.

**Table 1:** Socio-demographic characteristics of the incident chronic patient population by year between 2017 and 2020

	**2017**	**2018**	**2019**	**2020**
Incident chronic consumer population	46 308	47 684	47 931	77 222
Age (mean; standard deviation)	67.6; 15.6	68; 15.7	68.2; 15.6	66.4; 15.7
Age (median, interquartile range)	69; 22	69; 22	70; 22	68; 21
Gender (male) (n, %)	20 562 (44.4%)	22 205 (46.6%)	21 812 (45.5%)	35 397 (45.8%)
General population in Pays de la Loire	3 757 600	3 781 420	3 800 000	3 801 800
Incidence rate of PPI chronic user	1.2%	1.3%	1.3%	2%

The characteristics of the prevalent chronic user population are described in Table 2.

**Table 2:** Socio-demographic characteristics of the prevalent chronic patient population by year in 2017 and 2020

	**2017**	**2018**	**2019**	**2020**
Prevalent chronic patient population	156 721	160 498	164 721	167 751
Age (mean, standard deviation)	70.6; 14.6	70.7; 14.6	70.9; 14.5	71.1; 14.4
Age (median, interquartile range)	71; 20	72; 20	72; 20	72; 20
Gender (Male) (n, %)	71 381 (45.5%)	73 033 (45.5%)	76 207 (46.3%)	77 775 (46.4%)
General population in Pays de la Loire	3 757 600	3 781 420	3 800 000	3 801 800
PPI chronic user prevalence rate	4.2%	4.2%	4.3%	4.4%

Among the prevalent chronic users, the average age over the four years was 70.8 years. The proportion of women was 54%. The prevalent patient population increased from year to year to reach 167,751 patients in 2020. The rate increased from 4.2% to 4.4% between 2017 and 2020.

The evolution of chronic PPI consumption between 2017 and 2020 is described in Table 3. The number of deaths (** +**) was considered in the calculation. The percentage of chronic treatment discontinuation in 2017 was 12.47% and remained stable to reach 12.37% in 2020.

**Table 3:** Evolution of chronic PPIs consumption over the study period

Year	Number of chronic patients as of January 1st of the year	Number of chronic patients who no longer use PPI from November 1 to December 31 of the year	Proportion of chronic treatment discontinuation for the year
2017	156 721	19 551 (52** +**)^*^	12.47%
2018	160 498	20 761 (71** +**)^*^	12.89%
2019	164 721	21 301 (95** +**)^*^	12.89%
2020	167 751	20 809 (131** +**)^*^	12.37%

^*^of which x died during the year but died before 31/10 of the year

Regarding the initiation of PPI therapy, in 2020, 87.1% of treatment initiations were made by general practitioners. Between 2017 and 2020, the number of GPs prescribing treatment initiations increased from 32.962 to 67.280 (+ 107%), involving 6318 to 9820 physicians respectively (+ 55%), and 61 to 122 for other medical specialties (+ 100%).

We were also interested in the specialty of the prescribing physician during the first refill of the chronic treatment, i.e. when a second prescription was used to refill the treatment. Between 2017 and 2020, there were 281,311 (10.5%) renewals by physicians practicing in health care institutions. General practitioners renewed 2,402,263 prescriptions (89.3%). The remaining 5221 (0.2%) were done by other medical specialties.

The International Nonproprietary Names (INN) of the PPIs and their dosage dispensed each year between 2017 and 2020 are described in Table 4. These data were for all chronic consumer patients. Between 2017 and 2020, esomeprazole 20 mg was the most dispensed drug among PPI chronic users.

**Table 4:** Distribution of PPIs dispensations between 2017 and 2020 in chronic consumer patients

PPI	Dosage	2017 (%)	2018 (%)	2019 (%)	2020 (%)
**ESOMEPRAZOLE**	**10 mg**	0.12	0.14	0.14	0.13
	**20 mg**	22.31	22.03	21.87	21.67
	**40 mg**	17.88	17.97	17.78	17.70
**LANSOPRAZOLE**	**15 mg**	6.75	7.11	7.70	8.18
	**30 mg**	5.84	6	6.21	6.44
**OMEPRAZOLE**	**10 mg**	3.66	3.69	3.54	3.49
	**20 mg**	14.73	14.66	14.14	13.78
**PANTOPRAZOLE**	**20 mg**	12.02	12.17	12.65	12.79
	**40 mg**	7.57	7.85	7.87	8.08
**RABEPRAZOLE**	**10 mg**	3.74	3.38	3.20	2.96
	**20 mg**	5.39	4.99	4.89	4.78

Chronic consumer patients had received a median of 336 DDDs per year between 2017 and 2020.

## Discussion

This study describes the trends of PPI prescription among chronic consumer patients in the Pays de la Loire region between 2017 and 2020. The percentage of PPI discontinuation among chronic users was stable over the 4 years of the study, around 12%. These results show a stagnation in the deprescribing of chronic PPI treatments in this region even though more and more studies are calling for awareness and strong PPI deprescribing [18]. In 2020, the French National Health Authority (HAS) published a reframing note and issued new recommendations on the proper use of this therapeutic class [7], after publishing a first report in 2009, updated in 2013 [19]. It seems that issuing recommendations alone is not sufficient to bend the prescribing curve of overused drugs, and that patient-centered interventions should be developed. Furthermore, no national validated algorithm for conducting and maintaining PPIs deprescribing has been issued in France, making it difficult to disseminate tools that can be used by all French health professionals. It therefore seems appropriate to create tools [20], and to implement targeted interventions aimed at reducing the consumption of patients chronically using PPIs, and maintaining the deprescribing [21]. The carrying out of such a study requires knowledge of the deprescribing evolution before the intervention (control arm), a result which is now available.

The definition of a chronic PPI user is not consensual, and has been found in the literature to span from 2 weeks to more than 7 years [22]. Yet, we chose a definition that was coherent with the current French guidelines, which suggest to reevaluate the treatment at 3 months. The threshold for classifying individuals as PPI chronic user or PPI non-chronic user was defined after looking for the change induced by using a temporal coverage of at least 75% or 85%. Considering a temporal coverage of 75% led to a 1% increase in the number of individuals classified as chronic PPI users, while considering a temporal coverage of 85%, reduced it by 1%. For a three months period, 80% of temporal coverage represented at least 74 days of PPI treatment. In France, the duration of PPI treatment for the main indications is less than 9 weeks, which represents a maximum of 56 days of PPI treatment, less than the threshold used (74 days) in our study.

In our study, the number of incident chronic drug users increased from year to year, with an incidence rate rising from 1.2% in 2017 to 2% in 2020 among the general population of the Pays de La Loire region (*p* < 0.0001). Similarly, by cumulative effect, the number of prevalent chronic drug users increased very slightly from 4.2% in 2017 to 4.4% in 2020 (*p* < 0.0001). Even though the sharp increase of the absolute number of incident chronic consumers in 2020 has no explanation to this day, this overall increasing trend in PPIs consumption is comparable to that observed in other European countries such as the United Kingdom or Spain, and confirms the global overconsumption of PPIs [23, 24].

The year 2020 was marked by the COVID-19 pandemic and its impact on global health systems. Due to the confinements and difficulties in accessing care during this period, a decrease in overall PPI consumption at the national level has been documented with a negative balance of -280,000 PPIs deliveries compared to expectation [25]. Still, our study has shown that the number of chronic users had not decreased, which means that the decrease concerned occasional users. Indeed, chronic users were protected by the adaptability of the French health system and by the creation of new opportunities. Even if there was a drop in physicians activity during containment (-23% for general practitioners; -46% for specialists; followed by a near return to normal thereafter), teleconsultations have experienced a real boom: they constituted 30% of the acts of private physicians at the height of the crisis [26]. In addition, pharmacists have had the possibility of renewal of chronic treatments without new prescription. This adaptability has allowed patients undergoing chronic treatment to avoid a break in care as much as possible, PPI included.

Between 2017 and 2020, esomeprazole was the most dispensed PPI among chronic consumer patients, with approximately 40% of the dispensed PPIs, vs 18% for omeprazole. This result differs from the French National Agency for the Safety of Medicine (ANSM) study for which omeprazole was the most prescribed PPI at a national level in 2015, with 37.4%, while esomeprazole reached 33.4% of dispensed boxes [6]. We have no explanation for this difference, given that the choice of molecule depends on the habits of prescribers.

Chronic PPI users received a median of 336 DDDs of PPIs per year. The consumption of a patient who would use PPIs daily for one year would be 365 DDD. Considering that approximately 12% of chronic users stopped each year, this figure of 336 DDD corroborates the chronic and daily consumption of our population.

Most of initiations and first renewals of chronic PPI treatment were prescribed by a general practitioner. Observational practice studies conducted in various French hospitals have shown that 30 to 60% of hospitalized patients were on PPIs at entrance and that of these prescriptions, only 16 to 40% complied with the Marketing Authorization indications [27]. In 20 to 50% of cases, the indication for the treatment was not known and was initiated before hospitalization [28]. These results confirm the predominant role of general practitioners in the prescription of PPIs, who are a privileged target of deprescribing actions.

The median age of incident chronic users was between 68 and 70 years. Thus, half of the patients initiating chronic treatment were younger. While several studies recommend targeting the elderly as a priority in future deprescribing interventions, it seems to us that it is wise to also focus on younger populations in future deprescribing interventions [8]. Although they are less at risk of polymedication, it is important to consider that even young populations are victims of over-prescription of PPIs, and that without intervention they could become the elderly consumers of the future.

The majority of prevalent and incident patients were women (around 54%), which is more than the proportion of women in the Pays de la Loire region (around 51.3%) [13]. These results are comparable to the ANSM study on national SNDS data, where users who initiated a PPI in 2015 were mostly women at 56.9% [29]. This can be explained in part by the fact that women are more likely to suffer from Gastro-Esophageal Reflux Disease according to Kim et al. [30]. In our study, it was not possible to know the indications for PPI treatments in chronic patients, making it difficult to assess the relevance of prescriptions.

On the other hand, the lack of data on the use of PPIs over the counter should be considered when interpreting the results. However, according to the ANSM in 2015, 90% of PPIs were dispensed in community pharmacies and 96.5% of the dispensed units were prescribed [6]. This limits the impact of non-prescription PPI dispensing on our study.

Because the sample results are not probabilistic of the French population, we cannot conclude that the results are generalizable to the entire country. Nevertheless, the strength of our study is the use of a large database of the Health Insurance, in a region whose socio-demographic composition is almost similar to that of the country. Because of these characteristics, it is highly possible that the situation is similar at the national level. Further studies should be conducted to verify this hypothesis. To our knowledge, this study is the first to measure the cessation of PPI use by chronically using patients over several years.

## Conclusions

The stagnation of the PPI deprescribing process over the last 4 years reinforces the interest of setting up a deprescribing project in a French context, jointly led by health professionals (physicians, pharmacists…) and patients themselves. Knowing now the deprescribing rate without intervention makes it possible to define a control arm for a future PPI deprescribing study. This provides a basis for comparison to assess the effectiveness of a deprescribing intervention. This intervention could be, for example, the establishment of protocols between general practitioners and pharmacists, or the implementation of motivational talks based on the idea that patient empowerment is fundamental in order to move towards a reduction or cessation of a drug that is chronically consumed, but whose benefit-risk balance is no longer favorable.

## Data Availability

According to data protection and the French regulation, the authors cannot publicly release the data from the French national health data system (SNDS). However, any person or structure, public or private, for-profit or nonprofit, is able to access SNDS data upon authorization from the French Data Protection Office (CNIL Commission Nationale de l’Informatique et des Libertés) to carry out a study, a research, or an evaluation of public interest (https://www.snds.gouv.fr/SNDS/Processus-d-acces-aux-donnees and https://www.indsante.fr/). CNAM has permanent regulatory access to the data from the French National Health Data System (SNDS) via its constitutive bodies DRSM-CNAM. This permanent access is given in accordance with the French Decree No. 2016–1871 of December 26, 2016 relating to the processing of personal data called the “National Health Data System”, and French law articles Art. R. 1461–13 and 14. All requests in the database were made by duly authorized people, all methods were carried out in accordance with relevant guidelines and regulations and the study was conducted in accordance with the Declaration of Helsinki. In accordance with the permanent regulatory access granted to DRSM-CNAM, this present work did not require the approval from the French Data Protection Authority (CNIL). The study was registered on the study register of DRSM Pays de la Loire—CNAM concerning studies from SNDS data. For any request, please contact the third author (PA).
